# Identification of a ferroptosis-related gene signature predicting recurrence in stage II/III colorectal cancer based on machine learning algorithms

**DOI:** 10.3389/fphar.2023.1260697

**Published:** 2023-08-30

**Authors:** Ze Wang, Chenghao Ma, Qiong Teng, Jinyu Man, Xuening Zhang, Xinjie Liu, Tongchao Zhang, Wei Chong, Hao Chen, Ming Lu

**Affiliations:** ^1^ Department of Epidemiology and Health Statistics, School of Public Health, Cheeloo College of Medicine, Shandong University, Jinan, China; ^2^ Clinical Epidemiology Unit, Qilu Hospital of Shandong University, Jinan, China; ^3^ Department of Gastroenterological Surgery, Shandong Provincial Hospital, Shandong University, Jinan, Shandong, China; ^4^ Clinical Research Center of Shandong University, Jinan, China; ^5^ Department of Gastrointestinal Surgery, Key Laboratory of Engineering of Shandong Province, Shandong Provincial Hospital Affiliated to Shandong First Medical University, Medical Science and Technology Innovation Center, Shandong First Medical University & Shandong Academy of Medical Sciences, Jinan, Shandong, China

**Keywords:** stage II/III colorectal cancer, machine learning, prognosis, ferroptosis-related gene, tumor heterogeneity

## Abstract

**Background:** Colorectal cancer (CRC) is one of the most prevalent cancer types globally. A survival paradox exists due to the inherent heterogeneity in stage II/III CRC tumor biology. Ferroptosis is closely related to the progression of tumors, and ferroptosis-related genes can be used as a novel biomarker in predicting cancer prognosis.

**Methods:** Ferroptosis-related genes were retrieved from the FerrDb and KEGG databases. A total of 1,397 samples were enrolled in our study from nine independent datasets, four of which were integrated as the training dataset to train and construct the model, and validated in the remaining datasets. We developed a machine learning framework with 83 combinations of 10 algorithms based on 10-fold cross-validation (CV) or bootstrap resampling algorithm to identify the most robust and stable model. C-indice and ROC analysis were performed to gauge its predictive accuracy and discrimination capabilities. Survival analysis was conducted followed by univariate and multivariate Cox regression analyses to evaluate the performance of identified signature.

**Results:** The ferroptosis-related gene (FRG) signature was identified by the combination of Lasso and plsRcox and composed of 23 genes. The FRG signature presented better performance than common clinicopathological features (e.g., age and stage), molecular characteristics (e.g., BRAF mutation and microsatellite instability) and several published signatures in predicting the prognosis of the CRC. The signature was further stratified into a high-risk group and low-risk subgroup, where a high FRG signature indicated poor prognosis among all collected datasets. Sensitivity analysis showed the FRG signature remained a significant prognostic factor. Finally, we have developed a nomogram and a decision tree to enhance prognosis evaluation.

**Conclusion:** The FRG signature enabled the accurate selection of high-risk stage II/III CRC population and helped optimize precision treatment to improve their clinical outcomes.

## Introduction

Colorectal cancer (CRC) is a common and deadly disease with 147,950 new cases estimated in 2020 (Siegel et al., 2020; Mi et al., 2023). Early detection through regular screening, effective treatment options such as adjuvant chemotherapy and targeted therapies, and promoting healthy lifestyle choices can all help reduce the CRC recurrence risk and improve survival rates (Dekker et al., 2019). The pathological staging at the time of diagnosis is a crucial determinant of both the recurrence risk and survival (Jeffery et al., 2019). Meanwhile, stage II/III CRC represents a significant proportion, accounting for about 70% of all CRC cases (Liu et al., 2022). Unfortunately, even with curative resection, 30–40% of the patients will experience recurrence, which can significantly impact their survival rates (Jeffery et al., 2019). More importantly, a survival paradox exists for patients with stage IIB/IIC and IIIA CRC, which cannot be well explained by traditional clinicopathological features or molecular signatures (Kim et al., 2015; Kim et al., 2019). Meanwhile, evidence in previous studies showed that patients who routinely received adjuvant chemotherapy after surgery did not respond equally even with the same stage (Shiovitz et al., 2014). Therefore, there is still a need to establish a novel recurrence-related prognostic model to identify the high-risk population of stage II/III CRC for clinical decision-making.

Recent studies have brought light to various molecular features in CRC that have been highly correlated with the prognosis and therapy response. Notably, these features include CMS classification, genomic alterations such as TP53, KRAS, and BRAF mutation, microsatellite instability (MSI), and tumor mutational burden (TMB), which have been recognized as relatively reliable biomarkers (Dienstmann et al., 2017; Dienstmann et al., 2019; Chong et al., 2022). The immunohistochemistry technique, particularly the multiplex immunohistochemistry or immunofluorescence (mIHC/IF) method, is frequently used to aid in pathology diagnosis as it reduces inter-observer variability and has the ability to label multiple markers per tissue section. However, it is important to point out that one potential disadvantage of mIHC is that the number of markers that can be simultaneously labeled is typically limited, usually between 3 and 7, which may not capture the full complexity of the biomolecular interactions underlying the disease pathology (Tan et al., 2020). It is worth mentioning that the CMS classification, which employs bulk RNA-seq data to stratify CRC patients into four subtypes, has emerged as a highly effective tool for identifying strong prognostic effects for both recurrence and survival, warranting further attention and analysis (Guinney et al., 2015; Stintzing et al., 2019).

Several molecular models have been developed to predict the recurrence and survival of stage II/III CRC, including lncRNA, hallmark-based, immune-based, methylation-based, and epithelial–mesenchymal transition (EMT)-related signatures, among others (Li et al., 2020a; Chong et al., 2021; Liu et al., 2022; Ren et al., 2022; Li et al., 2023). Ferroptosis plays a critical role in the development of CRC through several mechanisms, such as the build-up of lipid peroxides, disruption of the balance between glutathione and glutathione peroxidase 4, and disturbances in iron homeostasis (Song et al., 2023). Accumulating evidence has shown that the induction of ferroptosis in CRC successfully eliminates cancer cells resistant to other modes of cell death (Wang et al., 2022a). Several studies also established ferroptosis-related gene (FRG) or lncRNA signatures to predict stage II/III CRCs recurrence or prognosis (Wu et al., 2021; Yu et al., 2021; Du et al., 2022). However, the performance of these molecular models in prediction was different, and several studies did not emphasize sufficient validation with multiple datasets and attempt at the multiple modeling algorithm. The modeling algorithm combination should be further fine-tuned, and the validation procedure for the signature should be intensified to improve the credibility of the model.

Accordingly, the aim of the present study was to construct an mRNA expression signature using FRGs to identify patients at risk of relapse via a 10-fold and bootstrap machine learning framework. The constructed signature was also validated in five independent datasets and compared with clinical traits and molecular features and published signatures. Sensitivity analysis was performed to test the performance of the signature. A nomogram and decision tree, which integrated clinical and molecular features with the signature, were established to improve clinical outcomes and guide clinical practice.

## Materials and methods

### Data collection and preprocessing

**FIGURE 1:**
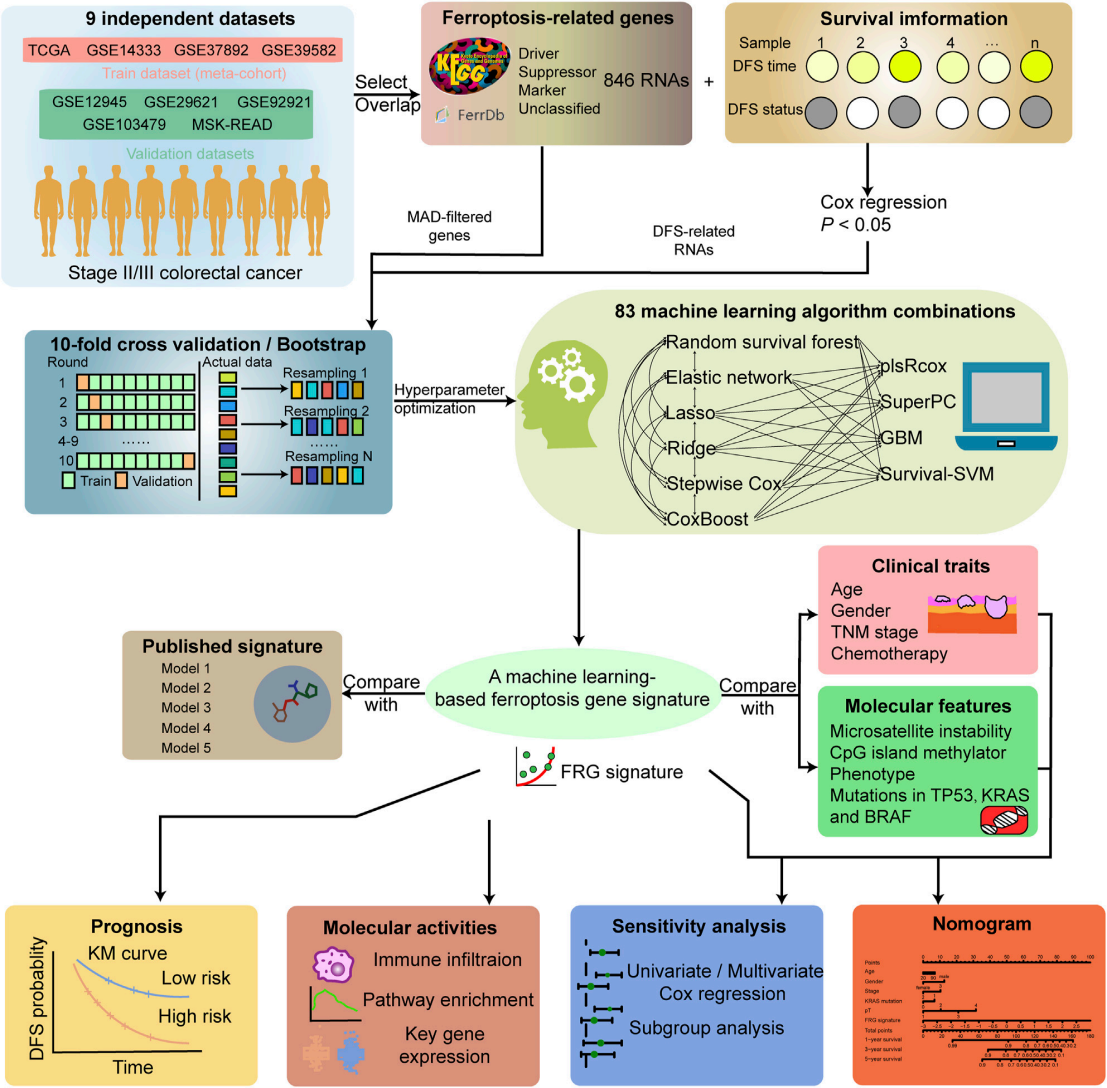
Workflow of the study.

The overall workflow of the study is shown in Figure 1. Gene expression data and corresponding clinical features of stage II/III CRC samples were collected from publicly available datasets of the NCBI GEO (https://www.ncbi.nlm.nih.gov/geo/), cBioPortal (https://www.cbioportal.org/), and TCGA (https://cancergenome.nih.gov/) database. Seven microarray datasets from GEO (GSE14333, GSE37892, GSE39582, GSE103479, GSE29621, GSE92921, and GSE12945) were sequenced by using Affymetrix HG-U133 Plus 2.0 Array, and TCGA (the combination of TCGA-COAD/READ) and MSK-READ datasets were produced from Illumina high-throughput sequencing platform. A total of 1,397 samples enrolled in the establishment and validation of the model met the following criteria: 1) primary tumor in colorectum; 2) with clinical information and gene expression data; and 3) stage II/III in the AJCC staging system.

Four cohorts (GSE14333, GSE37892, GSE39582, and TCGA-CRC, 1,000 samples) of the total patients were combined as the training dataset. The other five cohorts were used as independent validation datasets. The combined gene expression data in the training dataset was collected from the study of Guinney et al. (2015). Careful data preprocessing before the merge was implemented to address the batch effect among datasets due to the different platforms, labs, and time points. The raw data of the four GEO validation datasets were processed by the robust multiarray averaging (RMA) algorithm with ‘affy’ package, and the duplicate probes were merged via the median number. TCGA RNA FPKM format sequencing data were curated from UCSC Xena (https://xenabrowser.net/datapages/). TCGA-COAD/READ data were merged after removing the batch effect by Combat algorithm via “sva” package. Then, they were converted into TPM format and further log-2 transformed. The log-2 transformed RNA-seq data from the MSK dataset were collected from cBioPortal. All gene expressions were transformed into Z-score among samples when training the model.

### Acquisition and screening of ferroptosis-related genes

We obtained FRGs from the public databases FerrDb (http://www.zhounan.org/ferrdb/current/) and KEGG. A total of 846 RNAs were obtained, including those from the driver, suppressor, marker, and unclassified categories (Supplementary Table S1). After removing duplicates, a total of 546 RNAs remained. Since low-expressed or non-varying genes usually represent noise, the transcriptome data in the training dataset were downloaded with nearly 6,000 genes by the largest median absolute deviation (MAD). These genes were measured by at least one probeset in all datasets, and each gene was represented by the probeset with the largest MAD (Guinney et al., 2015). We took the intersection of the curated FRGs and MAD-filtered genes as the variable features in the training meta-cohort and performed univariate Cox regression analysis to screen out disease-free survival (DFS)-associated genes. A total of 80 representative recurrence-related ferroptosis genes were enrolled in the machine learning framework as initial variables (Supplementary Table S2).

### Construction of a prognostic gene signature

A total of 10 separate machine learning algorithms and their combinations composed the machine learning framework. The 10 algorithms included random survival forest (RSF), elastic network (ENet), least absolute shrinkage and selection operator (Lasso), ridge, stepwise Cox proportional hazards regression (Stepwise Cox), CoxBoost (Boosting Cox’s proportional hazards regression), generalized boosted regression modeling (GBM), supervised principal components (SuperPC), partial least squares regression for Cox (plsRcox), and survival support vector machine (survival-SVM). Six algorithms, including Lasso, RSF, CoxBoost, ridge, ENet, and stepwise Cox, could be used to perform feature selection. Within this framework, we utilize six specific algorithms for feature selection: Lasso, RSF, CoxBoost, ridge, ENet, and stepwise Cox. These algorithms play a fundamental role in the preliminary phase of gene screening, working synergistically with other algorithms. Both individual algorithms and combinations of two algorithms contribute to this comprehensive framework. To optimize the performance of each algorithm, we employed either 10-fold cross-validation (CV) or bootstrap resampling techniques. These approaches assist us in evaluating and honing the models. Finally, a total of combined 83 algorithms based on 10-fold CV or bootstrap resampling were utilized to select the optimistic performance model. The implementation of machine learning algorithms framework and corresponding hyperparameter optimization function are shown in Supplementary Table S3. The Harrell’s concordance index (C-index) was calculated among all datasets. The algorithm with the highest average C-index across all validation datasets was regarded as the optimal model to generate the FRG signature. The samples were categorized into high and low risk based on the optimal thresholds for signature scores determined by the surv_cutpoint function of the R package “survminer”.

### Collection of published signatures

To further assess our identified model’s performance, we curated five previously published mRNA signatures (Supplementary Table S4). These signatures were constructed by using diverse computational algorithms and curated from various biological processes, including hypoxia and the tumor microenvironment. To evaluate the performance of each signature, we employed univariate Cox regression analysis and computed the C-index across all cohorts.

### Construction and validation of the nomogram

A novel nomogram for predicting relapse in patients with stage II/III CRC was established by the “rms” package. We integrated common clinical and molecular features in the nomogram with our signature to compose a comprehensive model applying Cox proportional hazards regression. The samples in the training meta-cohort, which had these relevant variables (632 samples), were enrolled in the analysis. The calibration curve was used to visualize the relationship between the predicted probability generated by the nomogram and the actual observations. The decision curve analysis (DCA) results could be performed to obtain the clinical net benefit of different models, and all and none strategies (Van Calster et al., 2018). Finally, we employed recursive partitioning analysis with the R package “rpart” to construct a decision tree model for DFS, aiming to refine risk stratification.

### Additional bioinformatic analysis

The deconvolution approach xCell algorithm was selected to exhibit molecular features regarding immunology between risk groups by ‘xCell’ packages (Newman et al., 2015; Aran et al., 2017). xCell could utilize bulk RNA-seq data to infer infiltrating immune and stromal cell subsets. The correlation coefficients between the signature scores and each gene expression acquired were calculated. The sorted correlation coefficients were used as the ranked gene list input to perform gene set enrichment analysis (GSEA) via the “clusterProfiler” package against KEGG and REACTOME reference gene set (Subramanian et al., 2005).

### Statistical analysis

The data processing, statistical analysis, and plotting were generated in the R 4.2.2 software. The heatmap of genes enrolled in the signature with clinical annotations was generated using the R package “ComplexHeatmap.” Correlations between two continuous variables were evaluated via Spearman correlation coefficients. The Wilcoxon rank-sum test or *t*-test was applied to compare the difference between two groups for quantitative data. Two-sided Fisher exact tests were used to analyze categorical variables. The Cox proportional hazards model and Kaplan–Meier analysis were performed with the ‘survival’ or ‘rms’ package. Receiver operating characteristic curves (ROCs) were used to evaluate the prognostic classification performance of the signature with the ‘timeROC’ package. The C-index comparisons between clinical and molecular traits and the risk score were implemented by the “compareC” package. All statistical tests were two-sided. *p* < 0.05 was considered as statistically significant. The length of error bars represented 95% confidence intervals. The Benjamini–Hochberg method was applied to control the false discovery rate (FDR) for multiple hypothesis testing in appropriate conditions.

## Results

### Development of machine learning-based ferroptosis-related gene signature

**FIGURE 2:**
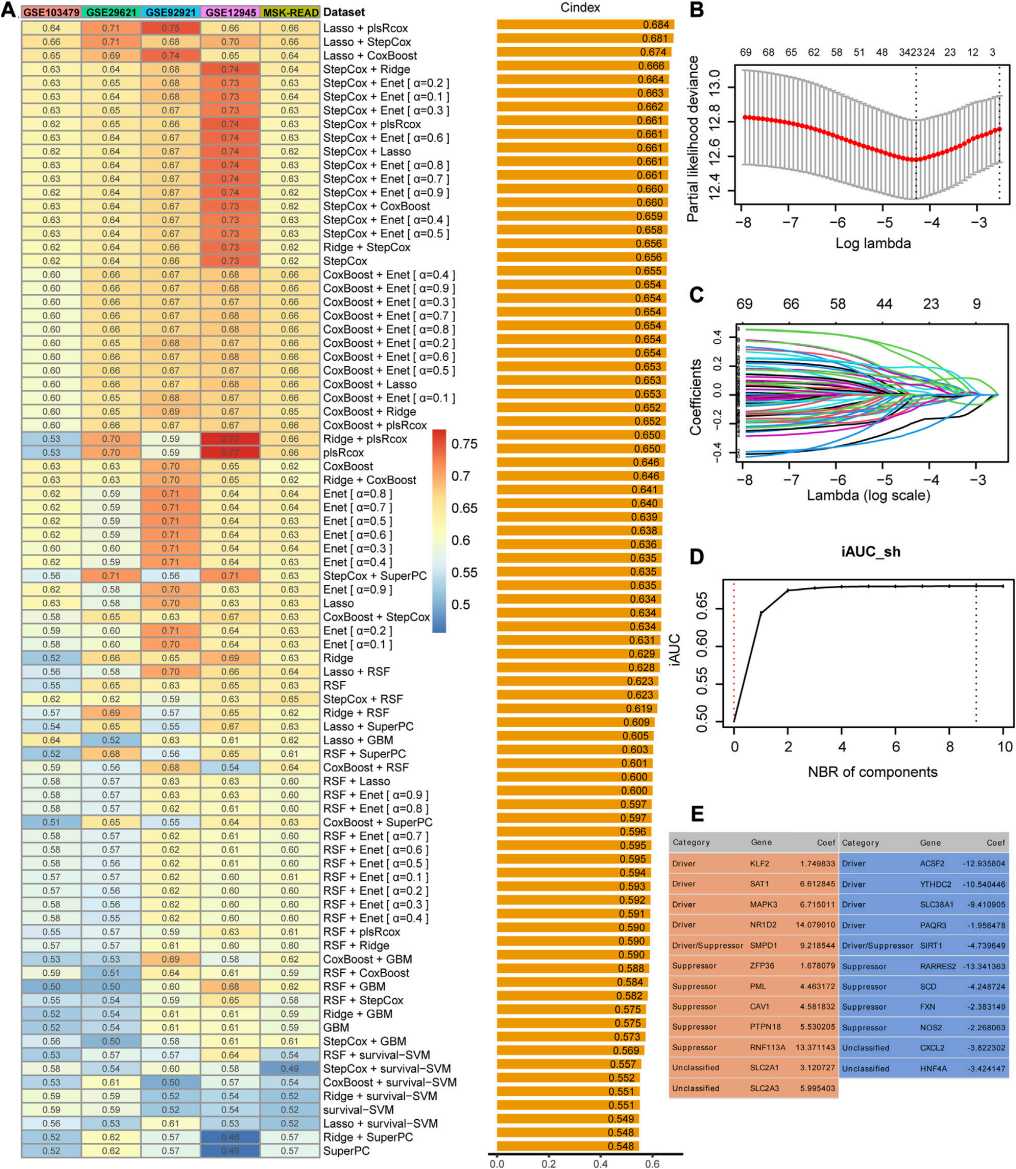
Identification and construction of the best performance signature. **(A)** C-indices of 83 combinations of machine learning prediction models in five validation cohorts. **(B)** Determination of the optimal lambda was obtained when the partial likelihood deviance reached the minimum value and further generated the gene features with non-zero coefficients. **(C)** Lasso coefficient profiles of the candidate genes for FRG signature construction. **(D)** Determination of the optimal number of components when the iAUC reached the maximum value. **(E)** Categories and coefficients of 23 genes finally obtained in plsRcox regression.

The meta-cohort of the four datasets (GSE14333, GSE37892, GSE39582, and TCGA-CRC) was regarded as the training dataset, and the principal component analysis showed no significant batch effects within the meta dataset (Supplementary Figure S1A). The expression profiles of the 80 ferroptosis-related prognostic genes were subjected to the machine learning-based modeling framework. We fitted 83 kinds of prediction models via 10 machine learning algorithms based on the 10-fold cross-validation or bootstrap resampling algorithm to optimize the model parameter in the training meta-cohort. The C-indices were calculated exclusively in the five validation cohorts of all models, and the model that exhibited the highest average C-index was deemed the optimal solution. The most robust model with the highest mean C-index in the five validation datasets was the combination of Lasso and plsRcox (Figure 2A). Using Lasso regression and 10-fold CV, we found that 23 FRGs had non-zero Lasso coefficients and were associated with recurrence in stage II/III CRC. The regression partial likelihood deviance reached its minimum value, indicating that these FRGs are important predictive features for recurrence (Figures 2B, C). The chosen features underwent a 10-fold cross-validation plsRcox to build a predictive model with the optimal number of components. The incremental area under the curve (iAUC) value reached its maximum at nine components (Figure 2D), so the model used components 0–9 to obtain fit statistics using both the Akaike information criterion (AIC) and Bayesian information criterion (BIC) (Supplementary Figure S1B). Finally, the risk score for each patient was calculated using the expression of 23 gene features multiplied by their corresponding coefficients to generate the FRG signature (Figure 2E).

### The predictive and prognostic value of FRG signature

**FIGURE 3:**
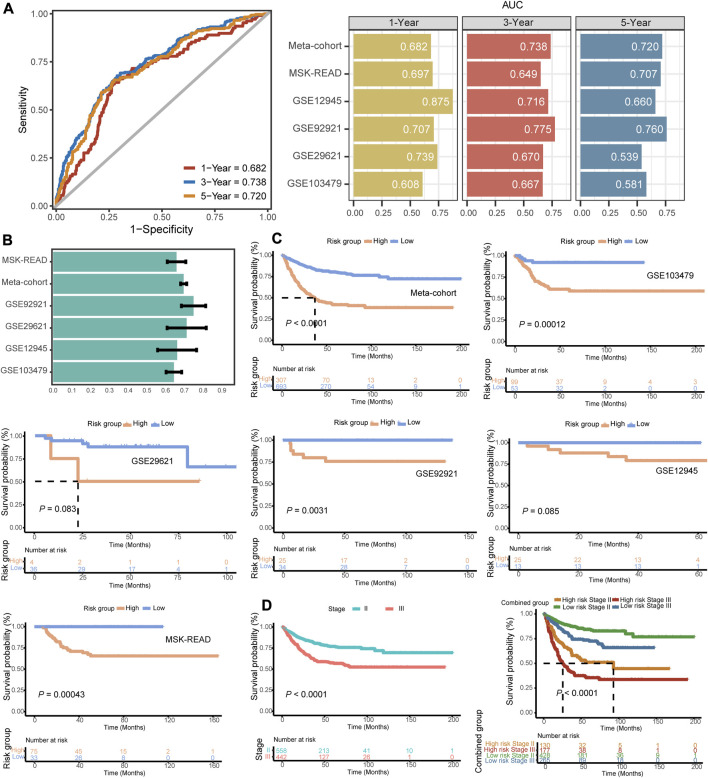
Evaluation indicators and prognostic value of the FRG signature. **(A)** Time-dependent ROC analysis for predicting DFS at 1-, 3-, and 5-year across the training meta-cohort and all validation datasets. **(B)** C-indices of the signature across all datasets. **(C)** Kaplan–Meier survival curve of DFS between patients with a high-signature score and with a low-signature score in the training meta-cohort and five validation datasets. **(D)** Kaplan–Meier survival curve of DFS between patients in stage II vs. stage III patients and with respect to the stage and the identified gene signature of the meta-cohort.

We used ROC analysis to measure the DFS discrimination of the signature, with 1-, 3-, and 5-year AUCs of 0.682, 0.738, and 0.720 in the training meta-cohort; 0.697, 0.649, and 0.707 in GSE103479; 0.875, 0.716, and 0.660 in GSE12945; and 0.707, 0.775, and 0.760 in GSE92921, respectively (Figures 3A, B). The 3-year AUC of GSE103479 and the 1-year AUC of GSE29621 were 0.670 and 0.739, respectively. The model had an overall certain decent degree of 3-year AUCs across all independent datasets. Both the training meta-cohort and validation datasets also showed stable C-indices around 0.7 (Figure 3C).

In our independent datasets, we divided the samples into high- and low-risk groups using the optimal cutoff for the predicted signature risk score. By analyzing the expression patterns of the 23 identified genes, we observed clear differences between the high- and low-risk groups (Supplementary Figure S1C). Specifically, genes such as ZFP36, KLF2, PML, PTPN18, MAPK3, SMPD1, SLC2A3, RARRES2, CAV1, and SAT1 were found to be highly expressed in the high-risk group. On the other hand, other genes showed predominant expression in the low-risk group. These findings suggest that these genes may play a significant role in distinguishing different risk groups in stage II/III CRC. Patients in the high-risk group had significantly shorter DFS compared to the low-risk group in the training meta-cohort (*p* < 0.0001), and similar trends were also observed in the validation datasets such as GSE103479 (*p* = 0.00012), GSE92921 (*p* = 0.0031), and MSK-READ (*p* = 0.00043) (Figure 3D). The results of Kaplan–Meier survival analysis in two cohorts, GSE29621 and GSE12945, reached a marginal statistical significance (*p* = 0.083 and 0.085, respectively), considering the smaller sample size. Meanwhile, the discriminatory power of the FRG signature scores was similar to the hazard obtained through pathological staging. In both stage II and stage III subgroups, patients with high signature scores had significantly shorter DFS (*p* < 0.0001) (Figure 3E). In addition, we integrated patients into a pooled cohort containing the training and validation cohorts to revalidate the prognostic value. The pooled cohort still showed a significant difference in DFS between the high- and low-risk groups (*p* < 0.0001), and this difference was also observed within both stage II and stage III subgroups (*p* < 0.0001) (Supplementary Figures S2A–C). Totally, the FRG-based model provided the promising potential in predicting the recurrence risk of stage II/III CRC.

### The comparisons with other features and collected signatures

**FIGURE 4:**
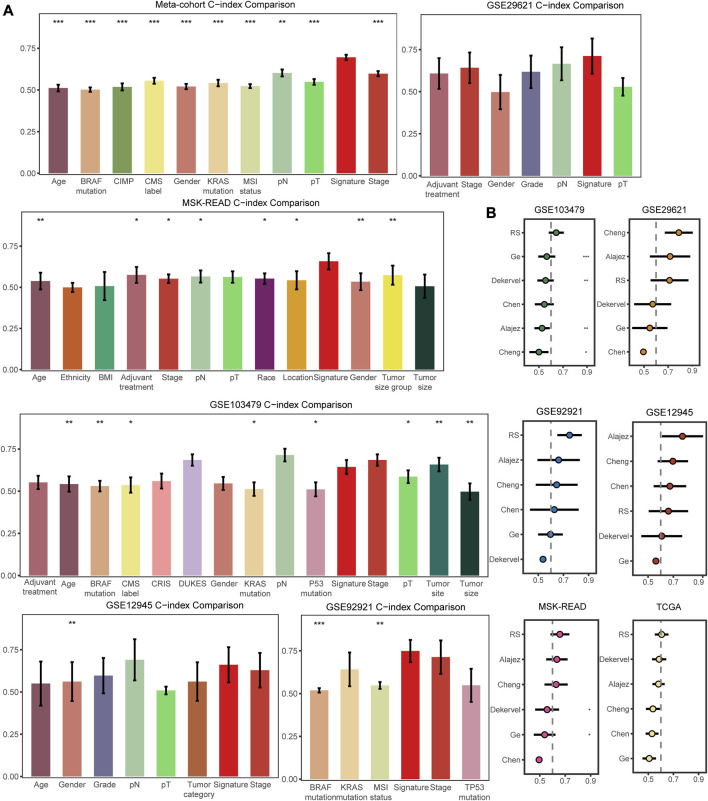
Comparisons of clinical and molecular characteristics, and published signatures with the FRG signature. **(A)** C-index comparisons between clinical and molecular variables and signature in the training meta-cohort and validation datasets. **(B)** C-index comparisons between signature and five published signatures. * means *p* < 0.05, ** means *p* < 0.01, and *** means *p* < 0.001.

Apart from selecting the most suitable model combination, we also compared the C-index of the signature with clinical characteristics and other molecular features in all the datasets included in our study. The clinical characteristics included demographic information, such as age, race, and gender, as well as tumor histology data such as AJCC stage, pT, pN, grade, tumor size, and chemotherapy response. Molecular features were specific to several mutational alterations (KRAS mutations, BRAF mutations, and microsatellite state) and molecular classification (such as CMS classification). These characteristics are commonly used in clinical evaluation of patients (Ng et al., 2019; Timar and Kashofer, 2020; Battistuzzi et al., 2021; Chen et al., 2021). The FRG signature score basically had the highest C-index compared to these clinical and molecular features in the training and validation cohorts, demonstrating the survival prediction capability of our signature (Figure 4A). Furthermore, our FRG signature achieved the highest C-index among five published molecular signatures in four datasets (Figure 4B). The multifaceted evaluation demonstrated that the FRG signature performed well in identifying stage II/III CRC patients with distinct clinical outcomes.

### Sensitivity analysis

**FIGURE 5:**
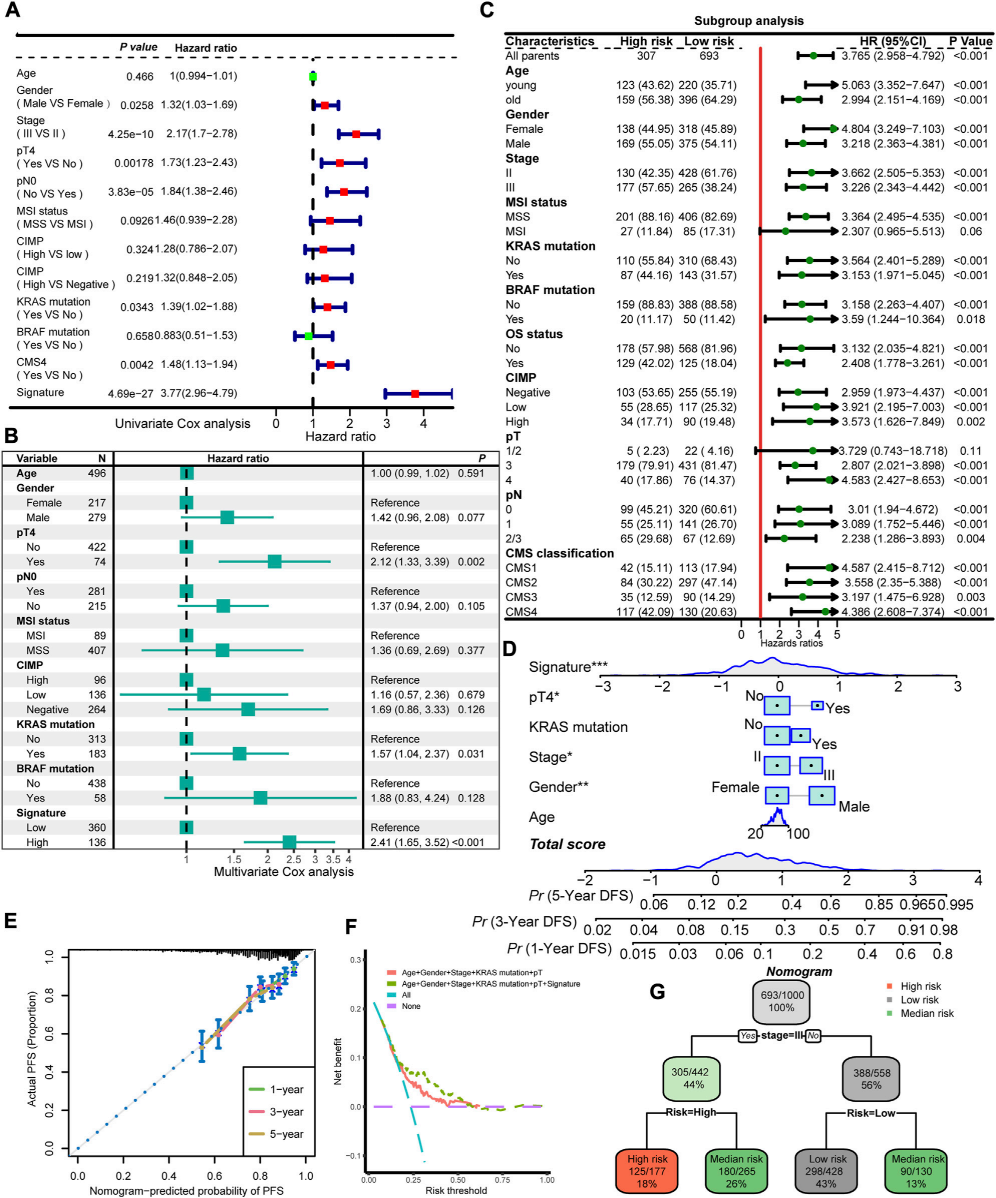
Interaction and combinations of the FRG signature with clinical and molecular features. Univariate Cox regression analysis **(A)** and multivariate Cox regression analysis **(B)** of prognostic factors for DFS for the training meta-cohort. **(C)** Subgroup analysis of the identified signature in clinical and molecular markers. **(D)** Prognostic nomogram predicting the probability of 1-, 3-, and 5-year DFS. **(E)** Calibration plot of the nomogram for 1-, 3-, and 5-year DFS prediction. Model performance is shown by the plot, relative to the 45-degree line, which represents perfect prediction. **(F)** DCA curve of the FRG signature and established risk factors in terms of DFS in the training cohort. The x-axis indicates the threshold probability, and the y-axis represents the net benefit. **(G)** A decision tree classifies patients into low-risk, intermediate-risk, and high-risk according to the probability of recurrent disease. * means *p* < 0.05, ** means *p* < 0.01, and *** means *p* < 0.001.

Table 1 summarized the distribution of demographics, tumor-related clinical characteristics, and molecular features of the four cohorts in the training meta-cohort, which included 1,000 patients identified as the high-risk group (N = 307) or low-risk group (N = 693) with the FRG signature. We recognized that stage, pN status, KRAS mutation, and CMS classification were significantly differentially distributed between high- and low-risk groups. To further inspect the robustness of the model, clinicopathological and molecular features, together with the identified signature, were assessed in univariate and multivariate models (Figures 5A, B). Importantly, only pT stage, KRAS mutation, and signature scores were independent recurrence prognostic factors in multivariable models. Gender, AJCC stage, and pN stage did not significantly improve prognosis prediction over pT stage and KRAS mutation when the FRG signature was considered. Sensitivity analysis showed that the FRG signature was still robust within the subgroups of clinicopathological and molecular annotation variables of interest, including pT4 and KRAS mutation (Figure 5C).

**TABLE 1:** Distribution of clinicopathological characteristics with low- and high-risk groups in the identified signature.

Variables	Level	Low	High	*p-*value
N		693	307	
Age (Sd)		67.380 (12.934)	66.897 (12.906)	0.6036
Gender (%)				
	Female	318 (45.89)	138 (44.95)	0.8373
	Male	375 (54.11)	169 (55.05)	
Stage (%)				
	II	428 (61.76)	130 (42.35)	<0.0001
	III	265 (38.24)	177 (57.65)	
pT (%)				
	1/2	22 (4.16)	5 (2.23)	0.232
	3	431 (81.47)	179 (79.91)	
	4	76 (14.37)	40 (17.86)	
pN (%)				
	0	320 (60.61)	99 (45.21)	<0.0001
	1	141 (26.70)	55 (25.11)	
	2/3	67 (12.69)	65 (29.68)	
MSI status (%)				
	MSI	85 (17.31)	27 (11.84)	0.0765
	MSS	406 (82.69)	201 (88.16)	
CIMP (%)				
	High	90 (19.48)	34 (17.71)	0.6528
	Low	117 (25.32)	55 (28.65)	
	Negative	255 (55.19)	103 (53.65)	
KRAS mutation (%)				
	No	310 (68.43)	110 (55.84)	0.0027
	Yes	143 (31.57)	87 (44.16)	
BRAF mutation (%)				
	No	388 (88.58)	159 (88.83)	1
	Yes	50 (11.42)	20 (11.17)	
CMS label (%)				
	CMS1	113 (17.94)	42 (15.11)	<0.0001
	CMS2	297 (47.14)	84 (30.22)	
	CMS3	90 (14.29)	35 (12.59)	
	CMS4	130 (20.63)	117 (42.09)	

### Potential molecular processes associated with FRG signature

To explore possible underlying molecular mechanisms for the FRG signature, we utilized the xCell method to analyze the immune infiltration landscape with our signature. A total of 14 immune infiltrating cells were correlated with the FRG signature (Supplementary Figure S3A). Representative cells, T cells and B cells, scored significantly higher in the low-risk group than in the high-risk group, while the levels of endothelial cells and epithelial cells were significantly higher in the high-risk group. To gain the comprehensive biological mechanisms of the FRG signature, we used the correlation coefficients between two major gene sets (KEGG and REACTOME) and signatures to perform GSEA. The extracellular matrix, cell adhesion processes, and elastic fiber formation were found positively correlated with the signature score. On the contrary, base excision repair, cell cycle, and RNA processes were found negatively correlated with the signature (Supplementary Figure S3B). In addition, the expression of key ferroptosis-related genes was also compared. We found DHFR and CYB5R1 were highly expressed in low- and high-risk groups, respectively (Supplementary Figure S3C).

### Establishment of predictive nomogram and decision tree

To provide potential clinical tool for recurrence prediction, we attempted to establish a nomogram together with the clinical and molecular features, and our signature. Age, gender, and stage status are considered primary characteristics that are routinely obtained in clinical practice. In addition, the results of a multivariate Cox regression analysis demonstrated that the FRG signature, pT, and KRAS mutation remained statistically significant even after adjusting for other factors. As a result, these specific characteristics were utilized in the development of a nomogram model (Figure 5D). In the calibration analysis of the nomogram, the prediction lines for 1-, 3-, and 5-year survival probability were closely aligned with the ideal reference line, indicating the favorable performance of the nomogram (Figure 5E). The DCA showed that the predictive model with the FRG signature yielded a higher net benefit compared to traditional prognostic factors enrolled in the nomogram (Figure 5F). In order to refine risk stratification, a recursive partitioning analysis was conducted on the 1,000 patients with the known tumor stage and FRG signature, resulting in a classifier decision tree (Figure 5G). The corresponding complexity parameter (CP) value with a minimum CV error at the first level was used to prune the decision tree (Supplementary Figure S4). In this analysis, the stage and FRG signature were identified as key determinants. Specifically, patients with stage II and a low FRG signature were categorized as the new low-risk group, while those with stage III and a high FRG signature were labeled as the new high-risk group. Patients who failed to align with these specific classifications were assigned to the intermediate-risk group. Overall, this approach helped to optimize risk stratification for each patient based on their unique characteristics.

## Discussion

The tumor AJCC stage is still the most widely used biomarker in clinical practice to provide guidance for treatment (Yoshihara et al., 2013). CRC shows apparent tumor heterogeneity in prognosis and therapy response, even with the same stage (Srdjan et al., 2016). In addition, the five-year postoperative recurrence rate for patients with stage II/III CRC is approximately 10–30% (Osterman et al., 2020; Xu et al., 2020; Benson et al., 2021). It is controversial to give all of them identical adjuvant therapies, regardless of the tumor genetic and molecular heterogeneity. Ferroptosis is a regulated form of cell death that is driven by iron-dependent lipid peroxidation. It plays a critical role in various physiological and pathological processes (Cui et al., 2020). There is emerging evidence suggesting that ferroptosis may be involved in cancer progression and treatment response, which has led to the interest in exploring its potential as a prognostic biomarker (Zuo et al., 2022). Accordingly, it is reasonable for us to use comprehensive FRG signature to develop the prognosis model and recognize high-risk subpopulations.

Several ferroptosis signatures for CRC have been developed, demonstrating the prognostic significance. However, these signatures primarily rely on Lasso and multivariable Cox regression analyses, without considering the combination of multiple algorithms to determine the optimal solution and performing thorough evaluations of the models on the validation set to assess their performance (Shao et al., 2021; Wang et al., 2022b; Du et al., 2022; Feng et al., 2022). To address this limitation, it is important to consider the combination of multiple algorithms and perform thorough evaluations of the models on the validation sets to assess their performance. This can help identifying the optimal solution and improve the robustness and generalizability of the prognostic signature. Machine learning algorithms have the advantages in making accurate predictions based on bulk data and using these predictions to guide future research efforts (Greener et al., 2022). Therefore, we were able to maximize the predictive accuracy of our model while also ensuring rigor and reproducibility. Additionally, the use of multiple independent validation datasets allowed us to evaluate the generalizability of the model and its performance in diverse population groups. However, while machine learning algorithms have shown great potential in making accurate predictions, selecting the optimal algorithm for model fitting can be challenging. Simply relying on researcher preference may not yield the best results and can lead to inefficiencies. One approach to address this issue is to use standardized methods for algorithm selection and model fitting. In our study, we took advantage of the strengths of machine learning and curated 10 different algorithms commonly used in survival analysis to generate an FRG signature for predicting stage II/III CRC prognosis. Rather than relying on a single algorithm or researcher preference, we utilized a combination of algorithms to create a framework and highlighted the importance of careful validation in this process. We calculated the C-index in multiple independent validation datasets to identify the best-performing model for predicting CRC recurrence.

In our study, we recognized 23 ferroptosis-related prognostic genes determined by the combination of Lasso and plsRcox with the highest average C-indices in validation datasets. The identified gene signature includes several FRGs associated with different aspects of cancer development. SAT1 plays a key role in immune regulation and metabolic signaling pathways, and it has been closely associated with chemoradiotherapy resistance and disease recurrence (Mou et al., 2022). MAPK3, a component of the RAS/MAPK pathway, may promote ferroptosis while potentially inhibiting antitumor immunity (Sun et al., 2022). NR1D2 is a transcriptional repressor that has been implicated in the epithelial–mesenchymal transition (EMT), a process that is crucial for cancer metastasis (Tong et al., 2020). Its knockdown could potentially slow down cancer progression by inhibiting EMT. CAV1 is a protein that plays a role in various cellular processes, including endocytosis and signal transduction. It has been identified as a suppressor of ferroptosis, a form of regulated cell death, and its high levels have been associated with the poor prognosis in cancer patients. Therefore, targeting CAV1 could potentially enhance the effect of ferroptosis and inhibit cancer progression (Lu et al., 2022). PTPN18 stabilizes the MYC protein level, leading to the activation of the MYC-CDK4 axis and promoting CRC development (Li et al., 2021). YTHDC2 is a tumor suppressor gene that is typically expressed at high levels in normal tissues and at low levels in tumor tissues. It has been associated with immune infiltrating levels, suggesting a role in the immune response to cancer (Li et al., 2020b). PAQR3 has been shown to induce apoptosis and inhibit proliferation and invasiveness of cancer cells when its expression is restored (Yu et al., 2015). Therefore, strategies to restore PAQR3 expression could potentially have therapeutic benefits in cancer treatment. Overall, these findings highlight the complex interplay between identified FRG genes and proteins in cancer progression.

In addition to the AJCC stage, several current and emerging clinically relevant biomarkers, such as BRAF mutations, HER2 overexpression and microsatellite state, were utilized to guide therapy in stage II/III CRCs (Sveen et al., 2020). To further validate the performance of the model, we compared C-index between the common clinical and molecular features (e.g., age, gender, T, N, AJCC stage, TMB, microsatellite state, and TP53, KRAS, or BRAF mutations) and our signature. Aging, male gender, and late stages are considered risk factors of CRC (Baraibar et al., 2023). TMB, KRAS, BRAF, and TP53 mutations were associated with worse prognosis, while high microsatellite instability (MSI-H) is a favorable prognostic biomarker and has been suggested as the predictors of immunotherapy response (Ganesh et al., 2019; Koncina et al., 2020). Our signature had high C-index levels in our comparisons with these common features, and the AUC of the 1- or 3-year relapses survival was robust across all datasets. Importantly, these markers of interest were found to be significant for recurrence in the univariate test, and the signature remained strongly predictive even after adjusting for them in the multivariate model. Moreover, our model demonstrated consistent predictive performance across subgroups with different clinical and molecular characteristics, further highlighting the robustness of the FRG signature as a prognostic tool. Collectively, these findings demonstrated that our signature could be a promising biomarker for predicting the high-risk recurrence population of stage II/III CRC in clinical practice.

The remarkable potential of the FRG signature score lies in its ability to precisely stratify patients into distinct high- and low-risk subgroups. This consequential stratification provides tailored and timely guidance regarding adjuvant therapy, specifically for stage II/III CRC patients. The FRG signature emerges as an effective tool in identifying stage II/III patients who are particularly susceptible to recurrence. Patients classified as stage III with a high FRG signature exhibited markedly decreased prognosis in comparison to those in stage II with a low FRG signature, while the intermediate-risk group comprising high-risk stage II and low-risk stage III patients shows similar outcomes.

The relevant biological activities of the extracellular matrix and cell adhesion and endothelial and epithelial cells were enriched in the high-risk group. They have been implicated in fibrosis, inflammation, thrombosis, cell division, and metastasis (Lin et al., 2021; Matthews et al., 2022). Several tumor suppressor pathways (e.g., base excision repair and cell cycle) were also enriched in the low-risk group. Alterations in iron metabolism and oxidative stress, key processes involved in ferroptosis, can be influenced by ECM remodeling and cell adhesion. Changes in iron import, export, and storage, as well as the presence of reactive oxygen species, can be regulated by ECM-related signals and cell adhesion molecules, thereby affecting the occurrence and progression of ferroptosis (He et al., 2023). Among the FRGs that we have identified in the signature, CAV1 plays a crucial role in the efficient deposition of ECM by fibroblast-derived exosomes, ultimately promoting tumor invasion. The activation of SIRT1 has a positive impact on the expression of the major ECM components and helps to regulate ECM organization (Albacete-Albacete et al., 2020; Smith et al., 2022). T cells and B cells were found highly infiltrated in the low-risk group. T cells have emerged as powerful allies in the fight against cancer, while B cells play a crucial role by presenting tumor-associated antigens to T cells. Recent studies have revealed that activated CD8^+^ T cells can enhance ferroptosis-specific lipid peroxidation in tumor cells, and this increased ferroptosis contributes to the antitumor efficacy of immunotherapy (Wang et al., 2019). KLF2 in the FRG signature is a transcription factor that has been demonstrated to play a crucial role in regulating the quiescence and trafficking of T lymphocytes. SATB1 directs lineage-specific transcriptional programs in the thymus, thereby influencing the development of the primary T-cell pool (Kakugawa et al., 2017; Wittner and Schuh, 2021). In addition, B cells can produce antibodies that enhance antigen presentation to T cells or directly target and kill tumor cells. This dynamic cooperation between T cells and B cells has a positive clinical impact (Waldman et al., 2020; Fridman et al., 2021). We found DHFR and CYB5R1, critical genes in ferroptosis, were highly expressed in low- and high-risk groups, respectively. Blockade of DHFR, either genetically or pharmacologically, enhances the effectiveness of GPX4 inhibition in triggering ferroptosis (Zheng and Conrad, 2020). Ferroptosis can also be induced by incidental electron transfer facilitated by POR/CYB5R1 oxidoreductase (Yan et al., 2021). This suggests that therapeutic approaches targeting ferroptosis induction may achieve favorable outcomes in the high-risk group. Overall, the underlying molecular mechanism suggested the biological plausibility and reliability in predicting the prognosis of the signature.

The nomogram we finally built present excellent performance. The capability of the FRG signature was validated with the calibration curve and DCA. The DCA curve demonstrated that incorporating the FRG signature yielded greater net benefit improvement compared to the conventional prognostic evaluation system. The prediction lines of the calibration curve for 1-, 3-, and 5-year survival probability were also fitted with the ideal reference line. In decision tree analysis, the intermediate-risk group regrouped stage II patients with a high signature and stage III patients with a low signature, thus enhancing the rationalization of risk groupings for stage II/III CRC patients. These results reinforced the potential for the FRG signature to guide personalized treatment decisions, improve outcomes for patients with CRC, and exhibit usability in daily routine practice.

Some limitations must be underscored with the current study even though the results of our investigation were profound. First, although a total of 1,397 patients were included with both microarray and RNA-seq platforms, they were all from retrospective cohorts. The signature should be further validated in a prospective study. Second, our study concentrated on the scope of ferroptosis-related mRNAs. Using the combination of lncRNA and mRNA might generate a more robust signature, which could be explored in future research. Last, the nomogram has been developed and validated in a computational model. However, it requires further clinical trials to confirm its effectiveness in real-world clinical settings and to evaluate its cost-effectiveness.

Identifying specific molecular targets involved in ferroptosis opens up avenues for developing novel therapeutic interventions. By targeting these genes or biological pathways in the FRG signature, it may be possible to modulate or inhibit ferroptosis, leading to improved treatment strategies for CRC. This could include developing small-molecule inhibitors or therapeutic agents that selectively target ferroptosis-related pathways. Moreover, integrating these FRGs and pathways with other omics data, such as proteomics and metabolomics, has the potential to uncover novel biomarkers and therapeutic targets. By combining multiple layers of molecular information, we can further gain insights into the complex interplay between different biological processes and identify key molecular players that can be targeted for therapeutic intervention.

In conclusion, our analysis established a stable and powerful ferroptosis-related signature based on consensus machine learning algorithms by sequencing the data of genes. The performance of the signature has been validated in multiple independent datasets and in comparison with the common clinical and molecular features. Furthermore, the model had great implications in the prognosis, even in subgroup analyses and after adjusting for common clinical and molecular markers. Finally, the developed nomogram, utilizing the common features and the signature, can potentially be a valuable tool to categorize high-risk patients. These findings indicate that the FRG signature shows promise in aiding clinical decision-making and facilitating personalized therapy for stage II/III CRC patients.

## Data Availability

The datasets presented in this study can be found in online repositories. The names of the repository/repositories and accession number(s) can be found in the article/Supplementary Material.
