# Supplementary material for: Identification of a ferroptosis-related gene signature predicting recurrence in stage II/III colorectal cancer based on machine learning algorithms
Source: Front Pharmacol. 2023 Aug 30;14:1260697. doi: 10.3389/fphar.2023.1260697 (PMC10498388; doi:10.3389/fphar.2023.1260697)
Supplement: Supplementary file 2 [file DataSheet1.DOCX]

**Supplementary figures**


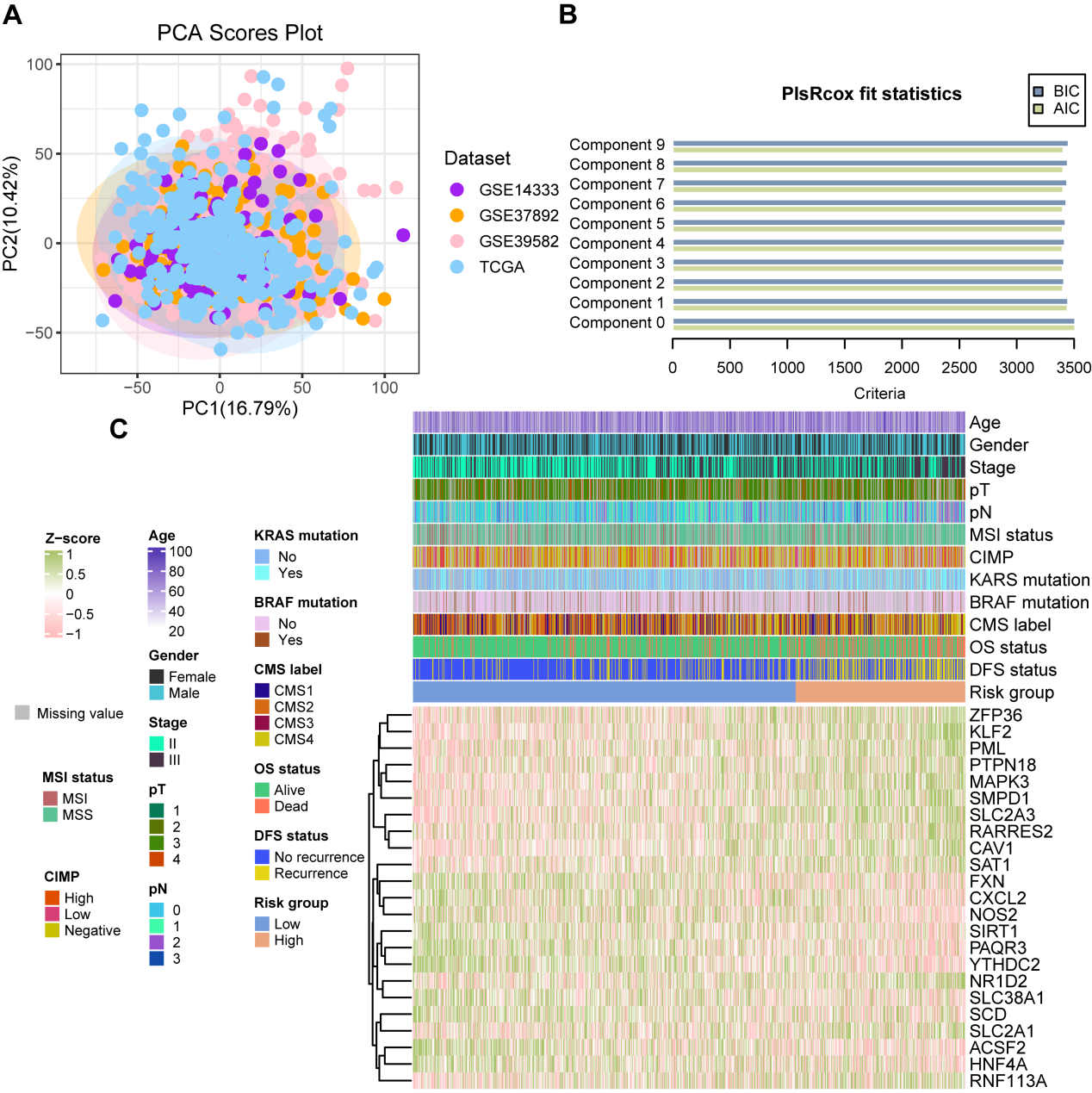


**Figure S1. Construction of machine learning-based ferroptosis-related gene signature.** (A) Principal component analysis of genes after the removal of batch effects in training meta-cohort from four independent cohorts. (B) Distribution of information criteria and fit statistics in plsRcox algorithm. (C) Heatmap revealing the differences of clinicopathological parameters in the high- and low-risk groups according to the expression patterns of the identified gene signature.

**
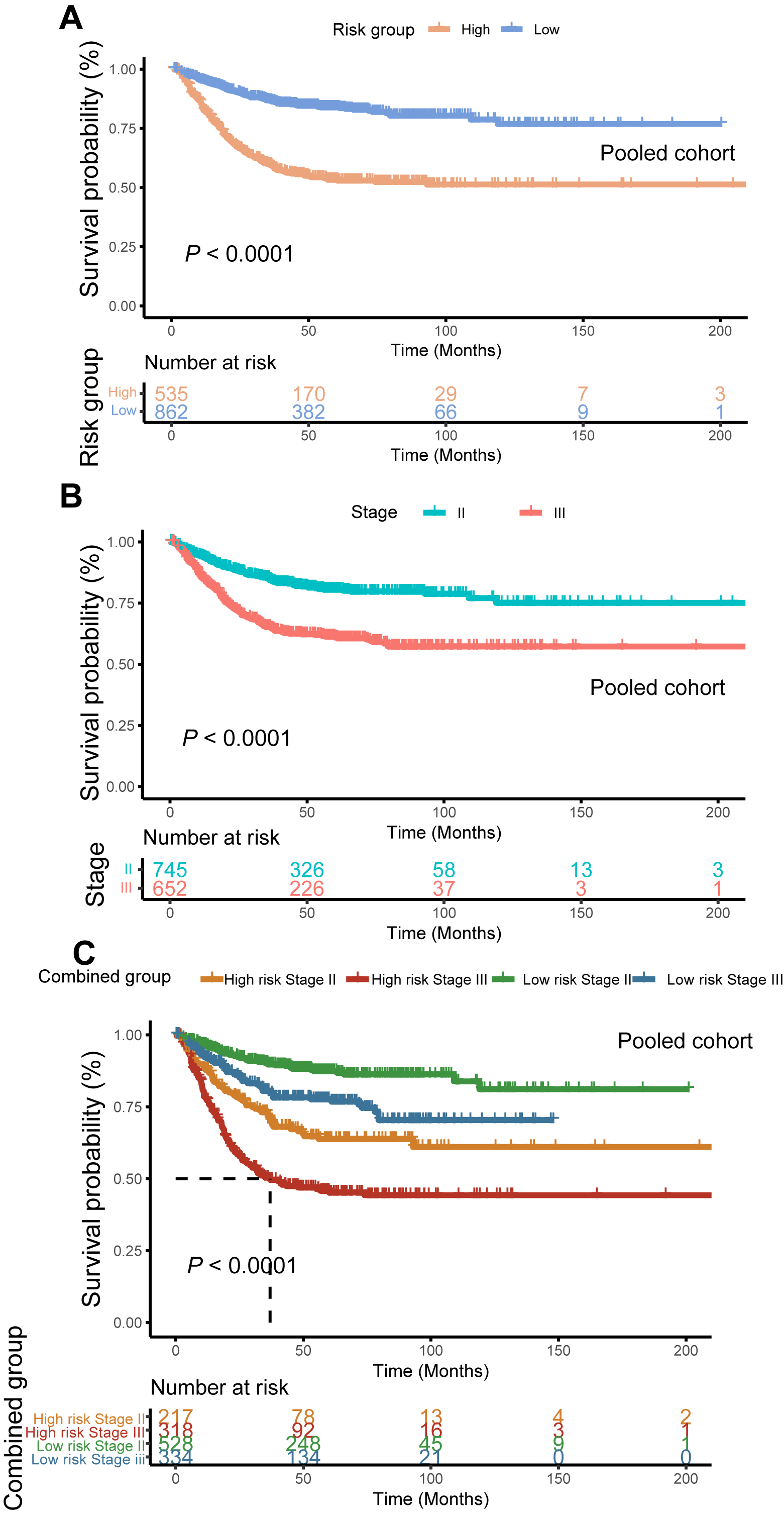
**

**Figure S2. Prognostic value validation in pooled cohort.** (A) Kaplan-Meier survival curves of PFS in high-risk patients versus low-risk patients of the pooled cohort. (B) Kaplan-Meier survival curves of PFS in stage II versus stage III patients of the pooled cohort. (C) Kaplan-Meier survival curves of PFS with respect to the AJCC stage and the identified gene signature of the pooled cohort.


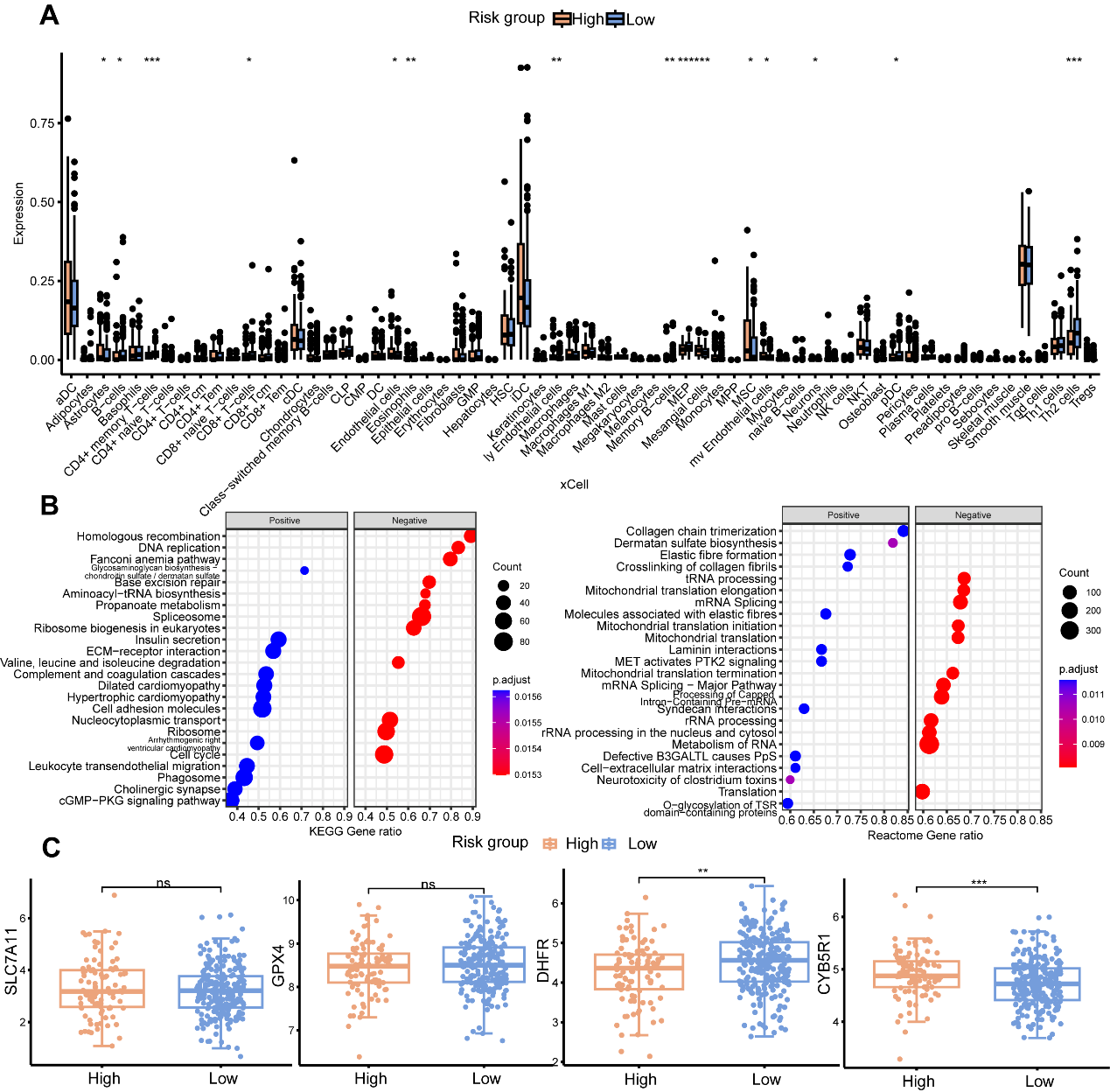


**Figure S3. Bioinformatics analysis of the identified signature.** (A) Relative distributions of 64 immune cells infiltration between high- and low-risk groups by xCell algorithm. (B) Dot plots of pathway enrichment that were positively and negatively correlated with identified signature against KEGG and REACTOME databases, respectively. (C) Relative distribution of ferroptosis biomarkers in SLC7A11, GPX4, DHFR and CYB5R1 between high- and low-risk groups. * means *P* < 0.05, ** means *P* < 0.01, *** means *P* < 0.001, ns means not significant.


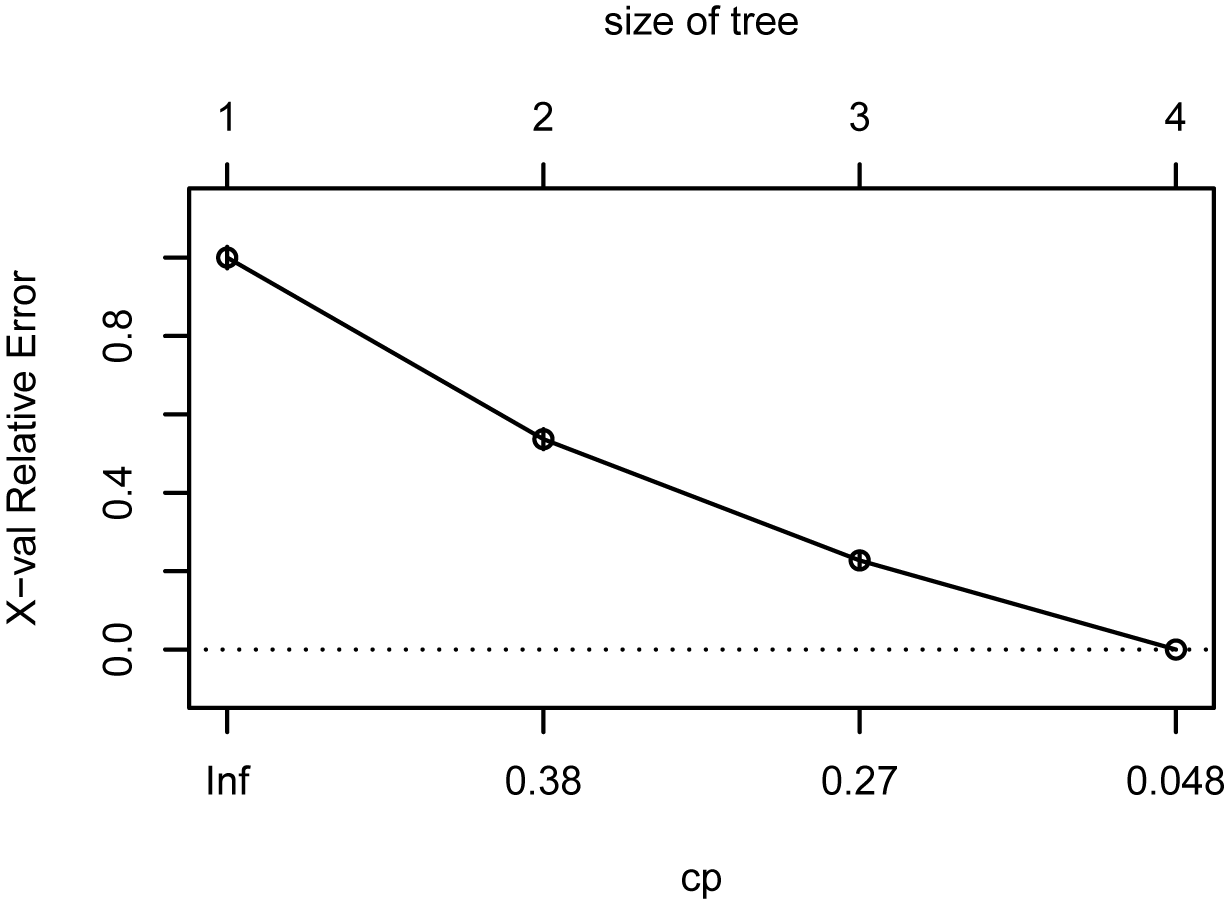


**Figure S4. The size of tree with minimum CP value used to build the decision tree model.**
